# In vivo ultraosound elastographic evaluation of the age-related change of human lens nuclear stiffness

**DOI:** 10.1186/s12886-020-01404-1

**Published:** 2020-04-06

**Authors:** Haiyan Zhou, Hong Yan, Weijia Yan, Xinchuan Wang, Qiaoying Li

**Affiliations:** 1 Department of Ophthalmology, Shaanxi Provincial People’s Hospital, Third Affiliated Hospital of the School of Medicine, Xi’an Jiaotong University, Xi’an, Shaanxi Province China; 2 Xi’an Fourth Hospital, Shaanxi Eye Hospital, Affiliated Xi’an Fourth Hospital, Northwestern Polytechnical University, Xi’an, 710004 Shaanxi Province China; 3 Medical School, The University of Sheffield, Western Bank, Sheffield, S10 2TN UK; 4 Basic Medical School, Air Force Medical University, Xi’an, Shaanxi Province China; 5 Department of UItrasonic Diagnosis, Tangdu Hospital, Air Force Medical University, Xi’an, Shaanxi Province China

**Keywords:** Ultrasound elastography, Strain rate ratio, Human lens nucleus, Stiffness, Age

## Abstract

**Background:**

To evaluate the age-related changes in the stiffness of the human lens nucleus in vivo.

**Methods:**

A total of 78 volunteers with best-corrected visual acuity of 20/20with a mean ± standard deviation intraocular pressure (IOP) of 16 ± 2.5 mmHg were divided into 3 groups of 26. The mean ages of Groups A, B and C were 81 ± 5.5, 44 ± 3.2 and 21 ± 2.5 years, with mean axial lengths of 23.8 ± 0.5 mm, 23.8 ± 0.4 mm and 23.9 ± 0.3 mm, respectively. Using an elastographer, the ultrasound echolucency and elastic strain rate of the lens nucleus of one eye, selected randomly, of each subject were measured three times. The strain rate of the lens cortex could not be assessed. The qualitative differences in the strain rates across the groups were assessed, and differences in the strain rate ratios of the lens nuclei across groups were analysed by one-way ANOVA.

**Results:**

The strain rates of the lens nuclei of Group A were much lower than those in Groups B and C, as assessed qualitatively; the elastograph images of the lens nuclei of the older group showed a blue colour.The strain rate ratios of the lens nuclei of Groups A, B and C were 0.02 ± 0.08, 0.69 ± 0.12 and 1.95 ± 0.85, respectively. The differences in the lens nucleus strain rate ratios across the groups were statistically significant, with *p*-values < 0.05.

**Conclusions:**

Ultrasound elastography demonstrated in vivo that an older age is associated with a statistically significantly lower lens nucleus strain rate ratio and therefore a markedly higher lens nuclear stiffness.

## Background

It is important to understand the material properties of the lens when investigating the age-related occurrence of cataracts and modelling the age-related decline in accommodative amplitude, which results in presbyopia. Although numerous studies have been conducted in which the elastic and shear moduli of the human lens were measured in vitro*,* there are few techniques that can be used to measure the material properties of the human lens in vivo*.*

Brillouin light scattering has been performed in vivo, and the results demonstrate that the longitudinal modulus, a measure of compressibility, of the human lens nucleusis greater than that of the lens cortex at all ages [1]. It was also found ex vivo that the longitudinal modulus is linearly related to the shear modulus, and therefore, the lens nucleus is less compressibleand stiffer than the lens cortex [1].This finding is supported by the results of multiple in vitro studies involvingconical probe indentation [2], shear rheometry [3], Brillouin light scattering [4] and the bubble-based acoustic radiation force technique [5].

The in vivo changes in the velocity of A-scan ultrasound waves within the lens have been used to detect nuclear cataracts [6] and to assess whether lens material properties change during accommodation [7].Optical coherence tomography has also been used to assess the lens nuclear stiffness [8]; however, these methods cannot be used to quantify the biomechanical material properties of the lens.

Non-invasive ultrasound elastography measurements of the strain rate [9, 10] and strain rate ratio [11] constitutea unique method for qualitatively and quantitatively evaluating the material properties of the human lens in vivo. However, the currently available device requires focusing on a region of interest that has essentially uniform elastic and shear moduli. Since the minimum diameter of the ultrasound beam is approximately 6 mm, only the biomechanical properties of the lens nucleus can be assessed.In the present study, the strain rate and strain rate ratio of the lens nucleus were evaluated in individuals in three different age groups.

## Methods

This prospective study was performed in the Department of Ophthalmology in Tangdu Hospital at the Air Force Medical University in China after the university institutional review board approved the study. Written and verbal consent for participation in the study was obtained from 38 males and 40 females between the ages of 19and 89 years. The 78 participants were divided equally into three separate groups of 26 by their age. In Groups A, B and C, there were 10 and 16, 12 and 14, and 13 and 13 females and males, respectively. The mean age and axial lengths of the participants in Groups A, B and C were 81 ± 5.5yearsand 23.8 ± 0.5 mm; 44 ± 3.2 years 23.8 ± 0.4 mm; and 21 ± 2.5 years and23.9 ± 0.3 mm, respectively. All participants had normal ophthalmic examination results except for cataractous changes in the older age group, Group A, with best-corrected visual acuity of 20/20 and mean IOP of 16 ± 2.5 mmHg.

Using a coupling agent, the 8 to 10 Hz ultrasonic probe of the elastographer (Model EUP2L 54 M, 7 L probe, Hitachi Ltd., Japan) was vertically aligned perpendicularly with constant pressure on the central anaesthetized cornea of the randomly selected eye. The participants were placed in the supine position and looked toward the ceiling with the contralateral eye. Once the ultrasound probe was positioned perpendicular to the anterior lens surface, the elastograher gave a signal, and an image of the lens was captured.The region of interest (ROI) was set to the minimum default size of approximately 6 mm so that only the strain rate of the lens nucleus was automatically calculated by the elastographer. Lens nuclear strain rates were recorded when the device was in the ultrasound echolucency and elastic imaging modes. In the elastic imaging mode, a pseudo-colour scale showed the relative strain rate of the lens nucleus. A blue-coloured lens nucleus implied a lower strain rate and therefore a stiffer lens nucleus than did green- or red-coloured nuclei. The mean and standard deviation of the strain rate ratios were calculated for each group, and the differences across groups were analysed by one-way ANOVA (SPSS Version 13).

## Results

**Fig. 1:**
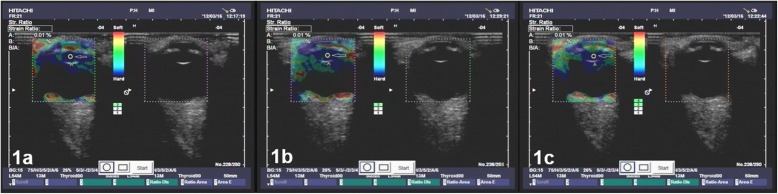
**a** to **c** Three images taken repeatedly with the ultrasound echolucency (right) and elastic (left) mode of the right eye of a 75 y/o man from Group A with a visual acuity of 20/20 and an axial length of 23.0 mm. The deep blue colour of the lens nucleus demonstrates that the strain rate is low, and therefore, the lens nucleus is stiff

**Fig. 2:**
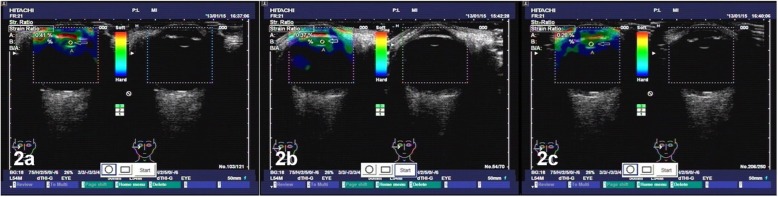
**a** to **c** Three images taken repeatedly with the ultrasound echolucency (right) and elastic (left) mode of the right eye of a 55 y/o woman from Group B with a visual acuity of 20/25 and an axial length of 23.1 mm. The dark green colour of the lens nucleus demonstrates that the strain rate is higher and therefore the lens nucleus is softer than those of the 75 y/o participant

**Fig. 3:**
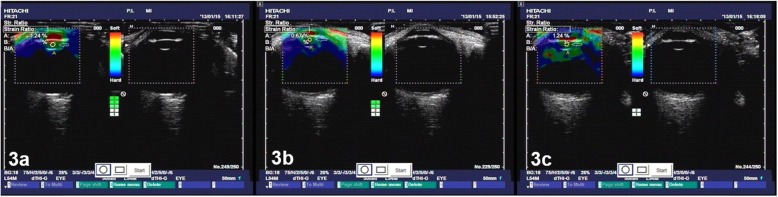
**a** to **c** Three images taken repeatedly with the ultrasound echolucency (right) and elastic (left) mode of the right eye of a 22 y/o man from Group Cwith visual acuity of 20/20 and an axial length of 23.7 mm. The dark green colour of the lens nucleus demonstrates that the strain rate and stiffness are approximately the same as those of the 55 y/o participant

**Fig. 4:**
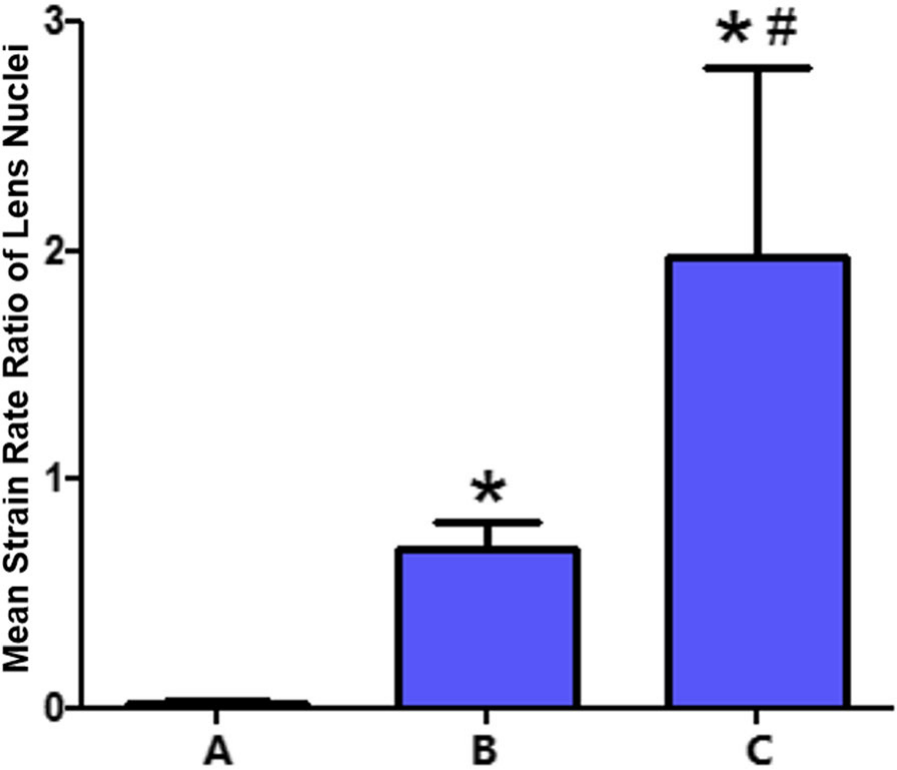
A graph of the mean and standard deviation (bars) of the lens nuclear strain rate ratios of Groups A (older age group), B (middle age group) and C (younger age group).Group C had a statistically significantly lower mean strain ratio than did Group B and Group C (*p*-values < 0.05); therefore, the lens nuclei of Group A were significantly stiffer than those of Groups B and C

The qualitative differences in the strain rates across the cornea, iris and lens nucleus are visible in the elastographic images, as shown in Figs. 1, 2 and 3.The cornea was green, and the iris was red independent of the participant’s age, demonstrating, as expected, that the iris is softer than the cornea and that the stiffness of the iris and cornea do not significantly change with age.In contrast, the lens nuclei of Groups A (older aged), B (middle aged) and C (younger aged) were dark blue, dark green anddark green, respectively.The strain rate ratios of the lens nuclei of Groups A, B and C were 0.02 ± 0.08, 0.69 ± 0.12 and 1.95 ± 0.85, respectively, as shown in Fig. 4. The differences in the lens nucleus strain rate ratios across the groups were statistically significant, with *p*-values < 0.05.

## Discussion

The elastographic strain rate ratio is definitively more strongly associated with the lens nuclear stiffness than is the elastographic strain rate. The lens nuclei in the middle and younger age groups had a similar green colour, making it difficult to discern differences in the strain rate between the two age groups (Figs. 2 and 3), while the d strain rate ratios were statistically significantly different. The lens nuclei in the middle age group had a strain rate ratio of approximately 1/3 that of the younger age group, demonstrating that the lens nuclei in the middle age group were stiffer. However, the minimal strain rate ratio of the older age group, which was approximately 35 and 100 times smaller than those of the middle and younger age groups, puts into perspective the marked stiffness of the lens nuclei in the older age group.This result in the lens nucleus is expected with octogenarians.

Ideally, the elastographer should be improved so that the ROI can be made smaller than a millimetre. This would permit us to determine the changes in the relative stiffness of the lens cortex and nucleus with age. It may also allow us to assess whether there are subtle differences in the stiffness within the lens nucleus. In this study, we examined only three specific age groups. In the future, a study of individuals of all ages should be conducted to establish the rate of change in the lens nuclear stiffness with age.

## Conclusions

Ultrasound elastography was used to demonstrate in vivo that an older age is associated with a significantly lower lens nucleus strain rate ratio and therefore a markedly higher lens nuclear stiffness. The elastographer strain rate ratio is a convenient and simple in vivo metric that can be used to quantitatively evaluate human lens stiffness. It may also be a valuable metric for assessing the biomechanical properties of all parts of normal and abnormal eyes.

## Data Availability

The data sets generated and analyzed during the current study are available from corresponding author on reasonable request.

## Abbreviations

IOP: Intraocular pressure
ROI: Region of interest
